# Acute Babesiosis Causing a False-Positive HIV Result: An Unexpected Association

**DOI:** 10.1155/2023/6271710

**Published:** 2023-07-24

**Authors:** Jody Z. He, Montasin Rezwan, Aneela Arif, Saada Baroud, Mohamed Elhaj, Aizaaz Khan

**Affiliations:** Flushing Hospital Medical Center, 4500 Parsons Blvd Queens, New York, NY 11355, USA

## Abstract

Babesiosis is a tick-borne condition that causes hemolytic anemia and manifests with flu-like symptoms such as fevers, chills, fatigue, and anorexia. Very few case reports have documented babesiosis infection associated with a false-positive HIV test. In this case report, we add to the current literature by describing a patient admitted for treatment of babesiosis who had a preliminary positive HIV test on admission and a negative repeat HIV test after one week of treatment for babesiosis. A 60-year-old male with a past medical history of high cholesterol presented to the Emergency Department after having abnormal laboratory tests with his primary care doctor. He reported fever, fatigue, anorexia, and worsening jaundice for three weeks. He was hypotensive and febrile on admission. A blood smear showed Babesia species with 1-2% infected red blood cells. He was admitted to the intensive care unit and received treatment with plasmapheresis, atovaquone, and antibiotics. The fourth-generation HIV 1/2 antigen/antibody test was initially positive but after treatment, HIV testing was negative. A misdiagnosis of HIV can greatly impact a patient's quality of life as antiretroviral therapy has multiple deleterious side effects. Clinicians must consider further evaluation of patients with acute babesiosis who also test positive for HIV.

## 1. Introduction

Babesiosis is a tick-borne disease caused by *Babesia microti*. It can cause fever, fatigue, chills, myalgia, and anorexia [1]. Patients who are immunocompromised can develop more severe symptoms such as intravascular hemolysis [2] and even have relapses of babesiosis after standard treatment [3]. Laboratory results can show hemolytic anemia, thrombocytopenia, and transaminitis, and a blood smear typically demonstrates Babesia species.

Here, we present a case of a false-positive HIV test in the setting of acute babesiosis. Although patients with HIV are more likely to have more severe presentations of acute babesiosis, few case reports document acute babesiosis associated with a false-positive HIV test. It has been posited that babesiosis can cause false HIV test results due to babesia and HIV serologies cross-reacting much like malaria and HIV serologies cross-react [4, 5].

We encourage repeat testing in patients who test positive for HIV in the setting of babesiosis in the appropriate clinical settings, for example, in patients without a history of HIV or without high-risk behaviors.

## 2. Case Presentation

A 60-year-old male with a past medical history of high cholesterol presented to the emergency department with intermittent fever, fatigue, anorexia, and worsening jaundice for three weeks. He was referred by his primary care doctor due to abnormal laboratory values and “parasites in the blood.” He reported that his urine had become dark yellow but there was no change in stool color. He denied any travel or hiking history. He worked for an HVAC (heating, ventilation, and air conditioning systems) repair company and stated that he occasionally needed to work in bushes outdoors though he did not recall any insect bites or rashes.

At presentation to the hospital, he was febrile with a temperature of 101.3°F, hypotensive at 94/55, and had an oxygen saturation of 95%. Pulse and respiration rate were within normal limits. The patient's white blood cell count was 3.9 K/*μ*L, the hemoglobin level was 6.2 g/dL, and platelet count was 19 K/*μ*L (Table 1). Prothrombin time and partial thromboplastin time were within normal limits. Alanine transaminase (ALT) was 51 U/L, aspartate transaminase (AST) was 87 U/L, and alkaline phosphatase was 257 U/L. Lactate dehydrogenase (LDH) was 3224 U/L, fibrinogen was >700 mg/dL, D-dimer was 15068 ng/mL, uric acid was 8.6 mg/dL, and reticulocyte count was 4.0%.

A blood smear showed *Babesia microti* with 1-2% infected red blood cells. Given the patient's severe presentation but relatively low percentage of infected red blood cells, HIV testing was done. The fourth-generation HIV 1/2 antigen/antibody test (Ortho Immunometric Technique (4th generation assay)) was preliminary positive, followed by a negative HIV-1 antibody result and an indeterminate HIV-2 antibody result. Viral hepatitis serologies, Lyme serology, and E. Chaffeensis antibody serologies were negative. A CT of his abdomen and pelvis showed hepatosplenomegaly without splenic infarct.

The patient was admitted to the medical intensive care unit for further management. He was treated with atovaquone and azithromycin for babesiosis with doxycycline to cover other possible tick-borne diseases. An exchange transfusion was performed due to the patient's severe presentation.

The patient's clinical condition improved over a week, and laboratory values of LDH, fibrinogen, ferritin, and uric acid began to normalize. At one week, his white blood cell count was 2.8 K/*μ*L, hemoglobin level was 8.1 g/dL, and platelet count was 249 K/*μ*L. A blood smear showed less than 0.1% of Babesia species. The patient had repeated HIV testing and HIV-1 RNA, which were negative. HIV-1 and HIV-2 viral loads were not tested. The CD4 count was 455. He was discharged home in good condition.

## 3. Discussion

This case report describes the case of a patient with acute babesiosis with severe symptoms who initially tested positive for HIV. After treatment and recovery from babesiosis, repeated HIV testing and HIV-1 RNA were negative. Complete blood counts improved as well with a notable increase in the reticulocyte count of 10.6% due to compensation from hemolysis.

A misdiagnosis of HIV can greatly impact a patient's health and quality of life. Patients who are positive for HIV must be treated with antiretroviral therapy which can have numerous side effects. Fourth-generation HIV testing is typically highly sensitive and has high clinical accuracy [6]. False-positive fourth-generation HIV tests are rare; a study of the results of fourth-generation HIV testing in low-risk populations showed a false-positivity rate of 0.09% [7]. However, there have been case studies that have reported that diseases such as schistosomiasis [8], chronic granulomatous disease [9], and even COVID-19 [10] have been associated with false-positive HIV test results. Few case reports have described false-positive HIV tests associated with acute babesiosis [2, 11]. Previous authors have speculated that false positivity in HIV could be due to Babesia serology interacting with HIV serology testing.

Patients with acute babesiosis who test positive for HIV on the fourth-generation HIV-1/2 antigen/antibody combination assay should undergo further investigation as recommended by the CDC HIV Testing Algorithm Guidelines to determine if the result is a true positive [12]. Follow-up testing includes HIV antibody differentiation and HIV RNA by nucleic acid amplification testing (NAAT). If HIV RNA by NAAT is negative, as it was in our patient, this identifies an initial false-positive result. Because the authors in this study reviewed the few case reports stating that false-positive HIV testing is possible in cases of acute babesiosis, antiretroviral therapy was not initiated at the time of the initial positive test, and the patient was not exposed to possible side effects of antiretroviral therapy as confirmatory testing was done.

While the reasons for false-positive HIV tests in acute babesiosis remain unclear, physicians who see patients with a positive HIV test in the setting of acute babesiosis should pursue further workup. Given the need for treatment of HIV, it is important that a physician exhibits due diligence and confirms the diagnosis. Our case report adds to the small amount of literature showing that false-positive HIV testing in patients with babesiosis is possible.

## 4. Conclusion

Acute babesiosis can yield a false-positive HIV test result, and patients with positive results should be further evaluated. Because antiretroviral therapy has multiple deleterious side effects, physicians should exhibit due diligence and confirm the diagnosis of HIV via confirmatory testing in the setting of acute babesiosis.

## Figures and Tables

**Table 1:** Laboratory values on the patient's day of admission and on the day of discharge.

Laboratory test	On admission	On discharge	Normal values
White blood cell	3.9	2.8	3.8–11 K/*μ*L
Hemoglobin	6.2	8.1	13.5–17.5 g/dL
Platelets	19	249	130–400 K/*μ*L
Alanine transaminase (ALT)	51	23	0–49 U/L
Aspartate transaminase (AST)	87	27	17–59 U/L
Alkaline phosphatase	257	136	38–126 U/L
Lactate dehydrogenase	3224	1354	313–618 U/L
Fibrinogen	>700	272	220–537 mg/dL
Ferritin	11700	1500	17.9–464 ng/mL
D-dimer	15068		<500 ng/mL
Uric acid	8.6	6.3	3.5–8.5 mg/dL
Reticulocyte count	4.0	10.6	0.5–2.0%

## Data Availability

All data generated or analyzed during this study are included in this article and are available upon reasonable request to the corresponding author.
